# Trends in the Asthma Burden Attributable to High Body Mass Index in Children Across 204 Countries and Territories, 1990–2021: An Analysis of the 2021 Global Burden of Disease Study

**DOI:** 10.1002/kjm2.70298

**Published:** 2026-10-12

**Authors:** Hua Li, Hai‐Yan Chen, Dong‐Zhi Zheng, Qiu‐Yi Liu, Jia‐Bing Li

**Affiliations:** ^1^ Department of Pediatrics Mianyang Maternal and Child Health Hospital Mianyang Sichuan China; ^2^ Department of Urology Mianyang Maternal and Child Health Hospital Mianyang Sichuan China

**Keywords:** childhood asthma, disability‐adjusted life years, high body mass index, mortality

## Abstract

High body mass index (BMI) has been identified as a significant risk factor for childhood asthma. Given the rising prevalence of childhood obesity, assessing the impact of high BMI on asthma burden trends is imperative. Utilizing Global Burden of Disease (GBD) data, we estimated age‐, sex‐, geography‐, and sociodemographic index (SDI)‐stratified asthma‐related deaths and disability‐adjusted life years (DALYs) attributable to high BMI in children (0–14 years old) (1990–2021). Health inequality was assessed by integrating SDI with the slope index of inequality and the relative concentration index. Bayesian age‐period‐cohort analysis projected asthma burden trends from 2022 to 2035. From 1990 to 2021, global asthma mortality due to high BMI declined, while DALYs rose. Females had higher mortality rates, whereas males exhibited greater DALY burdens. Mortality burden correlated negatively with SDI, whereas DALYs correlated positively. Cross‐national inequality analyses revealed higher mortality burdens in low‐SDI countries and elevated DALY burdens in high‐SDI regions. Frontier analyses indicated greater mortality reduction potential in low‐and low ‐ middle ‐ SDI areas and higher DALY mitigation opportunities in high‐SDI settings. Projections suggest a continued decline in asthma mortality but a rising DALY trend from 2022 to 2035. These findings highlight persistent sex‐ and SDI‐related disparities and support targeted strategies to reduce the global burden of childhood asthma attributable to high BMI.

## 1 Introduction

Asthma is a heterogeneous disease characterized by chronic airway inflammation and airway hyperresponsiveness, and it ranks among the most prevalent chronic respiratory diseases in children [1]. The global childhood asthma prevalence reached about 95.7 million cases in 2021, with an age‐standardized prevalence rate of 4758 cases per 100,000 [2]. This disease significantly impairs quality of life—nearly half of children with asthma cannot attend school regularly compared to their healthy peers [3], and their emergency department visits are nearly twice as frequent [4]. Childhood asthma imposes a substantial economic burden; in Australia alone, the average annual excess medical insurance costs associated with asthma treatment for children reach approximately AU$190.6 million [5].

High body mass index (BMI) ranks as the sixth leading risk factor for disability‐adjusted life years (DALYs) in the 2021 Global Burden of Disease (GBD) database [6]. Epidemiological evidence suggests that its adverse impact on asthma surpasses that of occupational asthmagens, making it a stronger risk factor for childhood asthma [7]. The systematic review indicated that maintaining a normal BMI trajectory during childhood can reduce the risk of developing asthma [8]. Global childhood obesity rates have risen dramatically—the prevalence of obesity alone among children and adolescents tripled from 1990 to 2021, with 93 million obese children aged 5–14 years in 2021, and this trend is projected to continue through 2050 [9]. Consequently, understanding the evolving burden of childhood asthma attributable to high BMI is imperative. Although existing studies have compared trends in the burden of childhood asthma attributable to high BMI between China and the United States [10], the systematic analyses of disparities across countries and regions with varying development levels worldwide remain scarce.

To address this gap, our study leveraged GBD 2021 data to comprehensively analyze trends in childhood asthma burden attributable to high BMI from 1990 to 2021 at global, regional, and national levels. We evaluated disparities by age, sex, and sociodemographic index (SDI) and projected trends through 2035. This work aimed to provide evidence for targeted prevention strategies and inform global efforts to mitigate childhood asthma.

## 2 Methods

### 2.1 Data Sources

Data were obtained from the GBD 2021 study (https://vizhub.healthdata.org/gbd‐results/, accessed February 14, 2025), which includes comprehensive disease burden data for 371 diseases and injuries and 88 risk factors across 204 countries and territories (covering 21 major global regions) from 1990 to 2021 [6, 11]. The GBD study integrates multiple data sources, including vital registration systems, verbal autopsies, population censuses, disease‐specific registries, and healthcare service data, to estimate disease burden via modeling approaches. All burden estimates are reported alongside 95% uncertainty intervals (UIs), which correspond to the 2.5th and 97.5th percentiles of 500 draws [11]. Data sources incorporated for each disease and risk factor can be accessed through the GBD Sources Tool (https://sources.healthdata.org/collection/sources‐2021).

To evaluate the input quality of cause‐of‐death data, the GBD calculates the Percent Well‐Certified (PWC) by integrating the completeness, representativeness, and cause‐of‐death coding quality of diverse cause‐of‐death data sources and applies a 0–5‐star rating system, where higher star ratings indicate better data quality. Based on the scores released by GBD 2021 [12], this study summarized the overall cause‐of‐death data quality for 204 countries and territories stratified by 2021 SDI quintiles (Table S1). High‐quality cause‐of‐death data are relatively limited in low‐SDI regions, and relevant estimates may be subject to substantial uncertainty.

This research extracted asthma‐related data attributable to high BMI in children aged 0–14 years globally and by sex (male and female) for 1990–2021, including: number of deaths, number of DALYs, crude mortality rate (CMR), crude DALYs rate (CDR), percentage change in CMR and CDR, age‐standardized mortality rate (ASMR), and age‐standardized DALYs rate (ASDR).

### 2.2 Definition

Asthma is a chronic lung disease characterized by bronchospasm, typically caused by allergic reactions or hypersensitivity, leading to breathing difficulties. Asthma was defined as wheezing diagnosed by a physician within the past year. The corresponding International Classification of Diseases (ICD)‐10 codes were J45 and J46, and the ICD‐9 code was 493 [11, 13].

The GBD study employs the Comparative Risk Assessment (CRA) framework to quantify disease burden attributable to risk factors. Based on well‐established risk factor‐outcome pairs, this framework calculates population attributable fractions (PAFs) by integrating risk factor exposure levels, relative risks (RRs), and theoretical minimum risk exposure levels (TMRELs), which are further used to estimate attributable disease burden. In GBD 2021, each risk‐outcome pair was modeled separately to estimate risk‐specific PAFs. Instead of calculating PAFs independently, this study directly adopted the estimates for the “high BMI‐asthma” risk‐outcome pair from GBD 2021. PAFs for other asthma risk factors not included in this study, such as air pollution and tobacco smoke, were estimated separately based on their corresponding risk‐outcome pairs. Detailed methodologies are described in previously published relevant literature [6].

The classification of high BMI in children and adolescents (under 18 years old) in GBD 2021 is based on the standards established by the International Obesity Task Force (IOTF). When age is measured in months, we used the monthly IOTF cut‐offs to determine overweight and obesity status. When age is measured in years, we used the midpoint of that year as the cut‐off [6, 14]. The specific IOTF cut‐off values can be found in Table S2. High BMI is defined as overweight and obesity according to age‐ and sex‐specific IOTF cut‐off values.

### 2.3 Statistical Analysis

#### 2.3.1 Descriptive Analysis

We compared the disease burden for asthma attributable to high BMI in children (all ages) between 1990 and 2021 at global, 5 SDI regions, 21 regions, and 204 countries and territories levels. Stratified analyses of disease burden trends were further performed across different sexes and age groups (0–4 years, 5–9 years, and 10–14 years). The estimated annual percentage change (EAPC) was calculated and adopted to evaluate temporal trends in CMR and CDR from 1990 to 2021.

#### 2.3.2 SDI Correlation Analysis

SDI is a composite indicator developed by the GBD team to measure the development level of countries or regions. It is calculated by taking the geometric mean of three core variables: the total fertility rate under age 25, the average years of education among people aged 15 and older, and lag‐distributed income per capita.

Based on SDI values, countries/regions are categorized into five tiers: high (0.810–1.000), high‐middle (0.712–0.810), middle (0.619–0.712), low‐middle (0.466–0.619), and low (0–0.466) [11, 15]. Spearman correlation analysis was used to examine associations between CMR, CDR, and SDI across 21 regions and 204 countries and territories.

#### 2.3.3 Cross‐National Inequality Analysis

Slope index of inequality (SII) and concentration index were utilized as two standard measures of absolute and relative gradient inequality, respectively, to quantify inequalities in asthma burden attributable to high BMI in children globally, across 21 GBD regions, and 204 countries and territories. SII was calculated by regressing national CMR and CDR against the relative position of the population ranked by SDI, with the scale defined by the midpoint of the cumulative population range [16]. The concentration index was calculated by numerically integrating the area under the Lorenz concentration curve, which was fitted using cumulative fractions of deaths and DALYs against the cumulative proportion of population ranked by SDI [17].

#### 2.3.4 Frontier Analysis

Frontier analysis was conducted based on the SDI to assess the potential room for improvement in the burden of childhood asthma attributable to high BMI among countries and regions with comparable developmental status. The frontier line consists of the attainable low disease burden levels corresponding to varying SDI values. For each country and region, the gap between its actual burden and the frontier benchmark was calculated as the “efficiency distance.” A larger efficiency distance indicates greater potential to reduce the burden of high BMI‐attributable childhood asthma [15, 18].

#### 2.3.5 Bayesian Age‐Period‐Cohort (BAPC) Prediction

The BAPC model was constructed using Integrated Nested Laplace Approximation (INLA) (23.09.09) and BAPC (0.0.36) R packages. This model employs first‐ or second‐order random walks to model age, period, and cohort effects within a Bayesian framework, thereby standardizing the influence of these effects [19]. We applied the BAPC model to predict ASMR and ASDR of asthma attributable to high BMI in children globally from 2022 to 2035.

All statistical analyses and visualizations were performed using R software (version 4.3.0).

## 3 Results

### 3.1 Trends in Global Asthma Burden Attributable to High BMI in Children, 1990–2021

#### 3.1.1 Global Level

From 1990 to 2021, both the number of global asthma deaths and CMR attributable to high BMI in children showed declining trends, while the number of DALYs and CDR demonstrated increasing trends. The number of deaths decreased from 640 cases (95% UI: 290–1059) in 1990 to 418 cases (95% UI: 206–657) in 2021, a reduction of 34.724%. During the same period, CMR represented a 43.572% decrease. The number of DALYs increased from 251,475 (95% UI: 114,889–455,594) to 344,739 (95% UI: 161,856–630,983), showing an increase of 37.087%. CDR exhibited an 18.505% increase (Table 1).

**TABLE 1:** Percentage changes in number of deaths, DALYs, CMR, and CDR for asthma attributable to high BMI in children at global level and across five SDI regions (1990 vs. 2021).

Location	Deaths number			DALYs number		
	1990 (95% UI)	2021 (95% UI)	Percentage change	1990 (95% UI)	2021 (95% UI)	Percentage change
Global	640 (290–1059)	418 (206–657)	−34.724%	251,475 (114,889–455,594)	344,739 (161,856–630,983)	37.087%
Low SDI	184 (74–335)	192 (86–327)	4.496%	36,716 (16,956–65,002)	72,235 (32,503–126,256)	96.737%
Low‐middle SDI	209 (95–351)	100 (50–155)	−52.170%	55,836 (25,348–99,788)	63,840 (29,823–114,379)	14.334%
Middle SDI	165 (77–273)	85 (45–131)	−48.420%	73,355 (32,839–133,969)	92,004 (41,173–170,431)	25.423%
High‐middle SDI	40 (19–62)	12 (6–18)	−69.608%	28,730 (12,756–52,222)	38,609 (16,400–73,905)	34.386%
High SDI	41 (20–64)	28 (15–41)	−33.319%	56,522 (25,227–104,790)	77,611 (36,794–145,136)	37.312%

Location	CMR/100,000			CDR/100,000		
	1990 (95% UI)	2021 (95% UI)	Percentage change	1990 (95% UI)	2021 (95% UI)	Percentage change
Global	0.037 (0.017–0.061)	0.021 (0.010–0.033)	−43.572%	14.460 (6.606–26.196)	17.135 (8.045–31.363)	18.505%
Low SDI	0.080 (0.032–0.146)	0.042 (0.019–0.071)	−48.024%	16.039 (7.407–28.396)	15.696 (7.062–27.433)	−2.144%
Low‐middle SDI	0.044 (0.020–0.074)	0.017 (0.009–0.027)	−61.057%	11.827 (5.369–21.136)	11.010 (5.143–19.726)	−6.908%
Middle SDI	0.029 (0.013–0.047)	0.015 (0.008–0.023)	−47.478%	12.708 (5.689–23.210)	16.230 (7.263–30.066)	27.714%
High‐middle SDI	0.014 (0.007–0.023)	0.005 (0.003–0.008)	−63.983%	10.500 (4.662–19.086)	16.722 (7.103–32.009)	59.257%
High SDI	0.022 (0.011–0.034)	0.016 (0.009–0.024)	−28.190%	30.420 (13.577–56.397)	44.983 (21.326–84.119)	47.873%

Abbreviations: CDR, crude DALYs rate; CMR, crude mortality rate; DALYs, disability adjusted life years; SDI, sociodemographic index; UI, uncertainty interval.

#### 3.1.2 SDI Level

Comparative analysis revealed differences in trends of asthma burden across regions with varying SDI levels. In 2021, the low‐SDI region exhibited the highest CMR at 0.042 per 100,000 population, whereas the high‐SDI region had the highest CDR of 44.983 per 100,000 population. From 1990 to 2021, CMR declined across all SDI strata. The high‐middle SDI region saw the largest reduction (−63.983%), while the high‐SDI region experienced the smallest drop (−28.190%).

By contrast, CDR decreased in low‐SDI and low‐middle SDI regions, while rising in all other strata, with the high‐middle SDI region showing the largest increase of 59.257% (Table 1).

### 3.2 Asthma Burden Attributable to High BMI in Children by Sex and Age

#### 3.2.1 Mortality Burden

Globally, the number of deaths and CMR of asthma attributable to high BMI among children aged 0–14 years declined for both males and females from 1990 to 2021. Although the death count was slightly higher in males in the early period, female deaths consistently surpassed male deaths after 1999, and females maintained a higher CMR throughout the study period (Figure 1A, Table S3). Stratified by age group, the overall mortality burden decreased among children aged 0–4 years and 5–9 years (Figure 1B,C, Table S3). By contrast, the total number of deaths did not fall in the 10–14 age group; despite a mild reduction in CMR, the magnitude of decline was smaller than that observed in the 0–4 and 5–9 year groups, and the gender gap in CMR widened over time (Figure 1D, Table S3).

**FIGURE 1:**
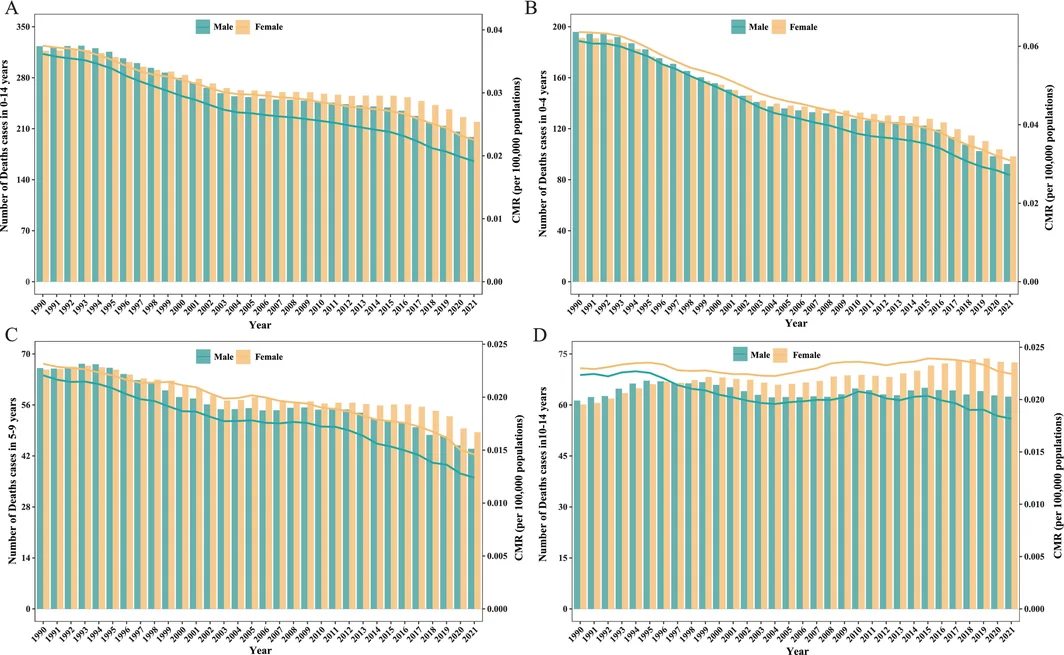
Global mortality burden of asthma attributable to high BMI in children by sex and age group, 1990–2021. (A) 0–14 years; (B) 0–4 years; (C) 5–9 years; and (D) 10–14 years. Bar charts show death numbers by sex and age group; lines represent CMR for males and females. CMR, crude mortality rate.

#### 3.2.2 DALYs Burden

From 1990 to 2021, the global number of DALYs and CDR of asthma caused by high BMI among children aged 0–14 years remained relatively stable in the early stage of the study period, followed by an upward trend after approximately 2005, with males consistently bearing a higher burden than females (Figure 2A, Table S3). Stratified by age group, DALYs and CDR first declined and then rose in children aged 0–4 years and 5–9 years. In contrast, the total DALY count kept increasing continuously in the 10–14 age group, whose CDR shifted from stable levels in the early years to a rising trend (Figure 2B–D, Table S3).

**FIGURE 2:**
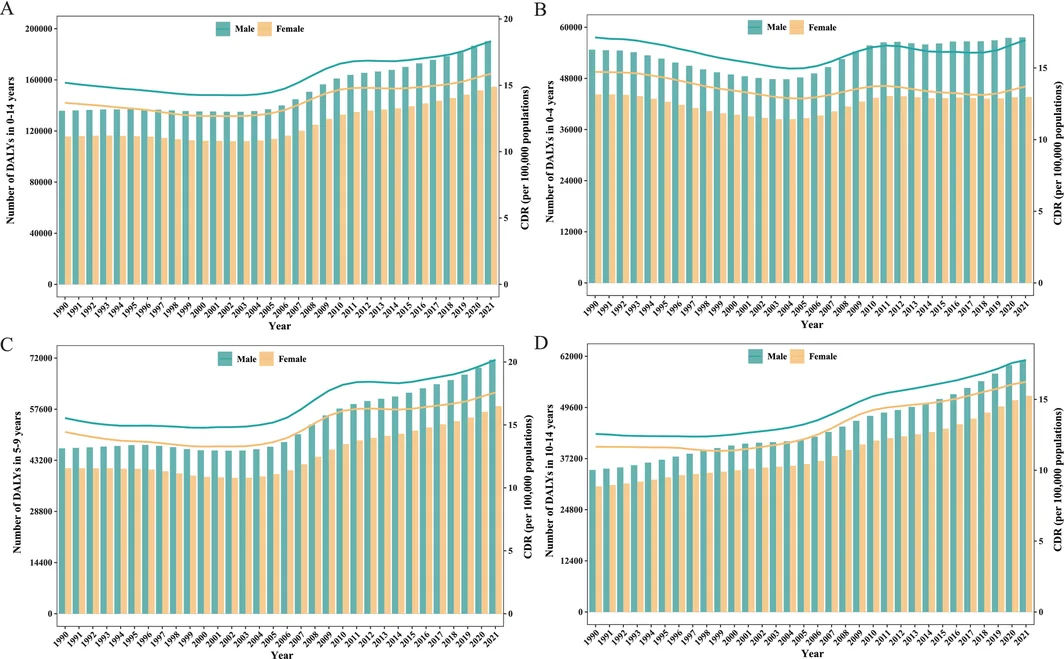
Global DALYs burden of asthma attributable to high BMI in children by sex and age group, 1990–2021. (A) 0–14 years; (B) 0–4 years; (C) 5–9 years; and (D) 10–14 years. Bar charts show number of DALYs by sex and age group; lines represent CDR for males and females. CDR, crude DALYs rate; DALYs, disability‐adjusted life years.

### 3.3 Asthma Burden Attributable to High BMI in Children Across 21 Regions and 204 Countries/Territories, 1990–2021

In 2021, substantial disparities in the burden of childhood asthma attributable to high BMI were observed across regions and countries. The Caribbean and Oceania had the highest CMRs, at 0.092 per 100,000 and 0.085 per 100,000, respectively. At the national level, Niue, Tokelau, and Kiribati ranked the top three in terms of CMR (Table S4). Regarding DALYs, High‐income North America led all regions in both total DALYs (46,570) and CDR (70.970 per 100,000), whereas South Asia had the lowest regional CDR at 4.520 per 100,000. Puerto Rico recorded the highest CDR (122.335 per 100,000), while Bangladesh, Pakistan, and Cambodia had the three lowest CDRs (Table S5).

EAPC analyses revealed that CMR declined in all regions except High‐income North America (EAPC = 0.214, 95% CI: −0.009 to 0.437). High‐income Asia Pacific exhibited the steepest CMR reduction (EAPC = −8.038, 95% CI: −8.342 to −7.732). Among all countries, Georgia saw the largest CMR drop (EAPC = −10.980, 95% CI: −13.040 to −8.872), and Zimbabwe had the most pronounced CMR increase (EAPC = 5.575, 95% CI: 4.199–6.970) (Figure 3A, Table S6). In contrast, CDR rose in most regions, with prominent annual growth exceeding 2% in Central Europe, East Asia, and Southern Latin America. By comparison, CDR decreased in Andean Latin America, Eastern Sub‐Saharan Africa, Southern Sub‐Saharan Africa, and Oceania. Across the 204 countries and territories, Poland, Taiwan (Province of China), and Oman showed the most striking CDR growth with EAPCs above 4% annually, while Guatemala experienced the greatest CDR decline (Figure 3B, Table S6).

**FIGURE 3:**
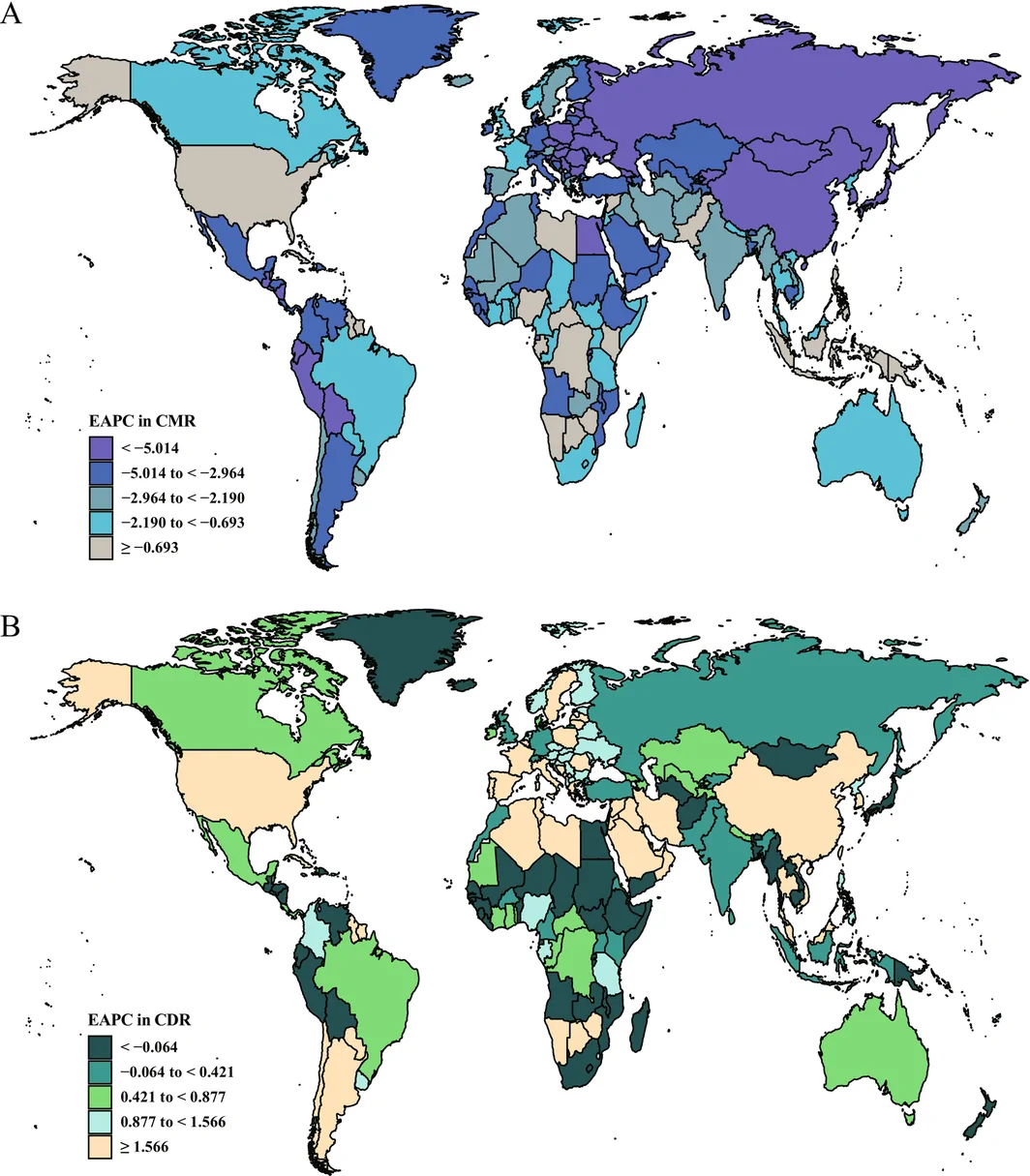
EAPC in CMR and CDR for asthma attributable to high BMI in children across 204 countries/territories, 1990–2021. (A) EAPC in CMR and (B) EAPC in CDR. Countries are color‐coded by quintiles of EAPC values for CMR and CDR: Darker to lighter colors represent increasing values. CDR, crude DALYs rate; CMR, crude mortality rate; DALYs, disability‐adjusted life years; EAPC, estimated annual percentage change.

### 3.4 Correlation Between Asthma Burden Attributable to High BMI in Children and SDI

From 1990 to 2021, CMR of childhood asthma attributable to high BMI was negatively correlated with SDI across the 21 regions (*ρ* = −0.330, *p* < 0.001), indicating a relatively higher mortality burden in regions with lower SDI (Figure 4A, Table S7). At the national and territorial level in 2021, CMR also displayed a negative correlation with SDI (*ρ* = −0.630, *p* < 0.001). While the CMR values of most countries and territories clustered around the fitted regression line, Niue and Tokelau presented markedly higher CMRs than predicted (Figure 4B, Table S8).

**FIGURE 4:**
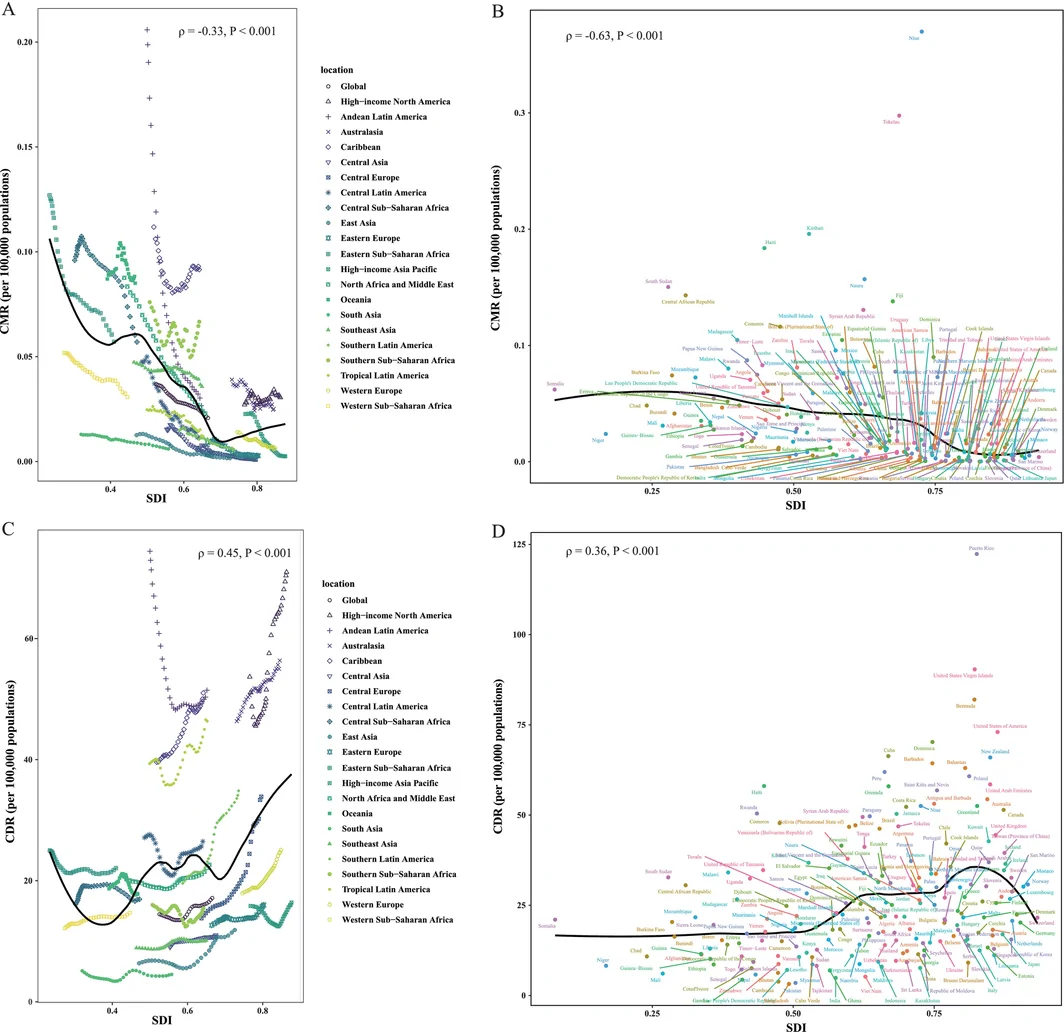
Trends and correlation analysis of asthma CMR and CDR due to high BMI in children in relation to SDI. (A) Trends in CMR and SDI globally and across 21 regions (1990–2021). (B) Trends and correlation between CMR and SDI across 204 countries and regions (2021). (C) Trends in CDR and SDI globally and across 21 regions (1990–2021). (D) Trends and correlation between CDR and SDI across 204 countries and regions (2021). (A and C) Different shapes represent the trends for the global level and 21 regions from 1990 to 2021. (B and D) Each dot represents a country or region. The black line indicates the fitted curve for the relationship between CR (CMR/CDR) and SDI. CDR, crude DALYs rate; CMR, crude mortality rate; DALYs, disability‐adjusted life years; SDI, sociodemographic index.

In contrast to CMR, CDR was positively correlated with SDI across the 21 regions from 1990 to 2021 (*ρ* = 0.450, *p* < 0.001) (Figure 4C, Table S7). A positive correlation was likewise observed at the national and territorial level in 2021 (*ρ* = 0.360, *p* < 0.001), with Puerto Rico showing a substantially higher CDR than expected (Figure 4D, Table S8).

### 3.5 Cross‐National Health Inequalities in Childhood Asthma Attributable to High BMI

Health inequality analyses revealed pronounced SDI‐related disparities in the global burden of asthma attributable to high BMI among children, with countries and territories at low SDI levels bearing a heavier CMR burden. The SII decreased from −0.090 (95% CI: −0.103 to −0.076) in 1990 to −0.042 (95% CI: −0.050 to −0.034) in 2021, indicating a reduction in absolute inequality for CMR. However, the concentration index shifted from −0.204 (95% CI: −0.309 to −0.099) to −0.306 (95% CI: −0.389 to −0.223), which demonstrated that CMR burden became further concentrated in low‐SDI settings, accompanied by widened relative inequality (Figure 5A,B, Table S9).

**FIGURE 5:**
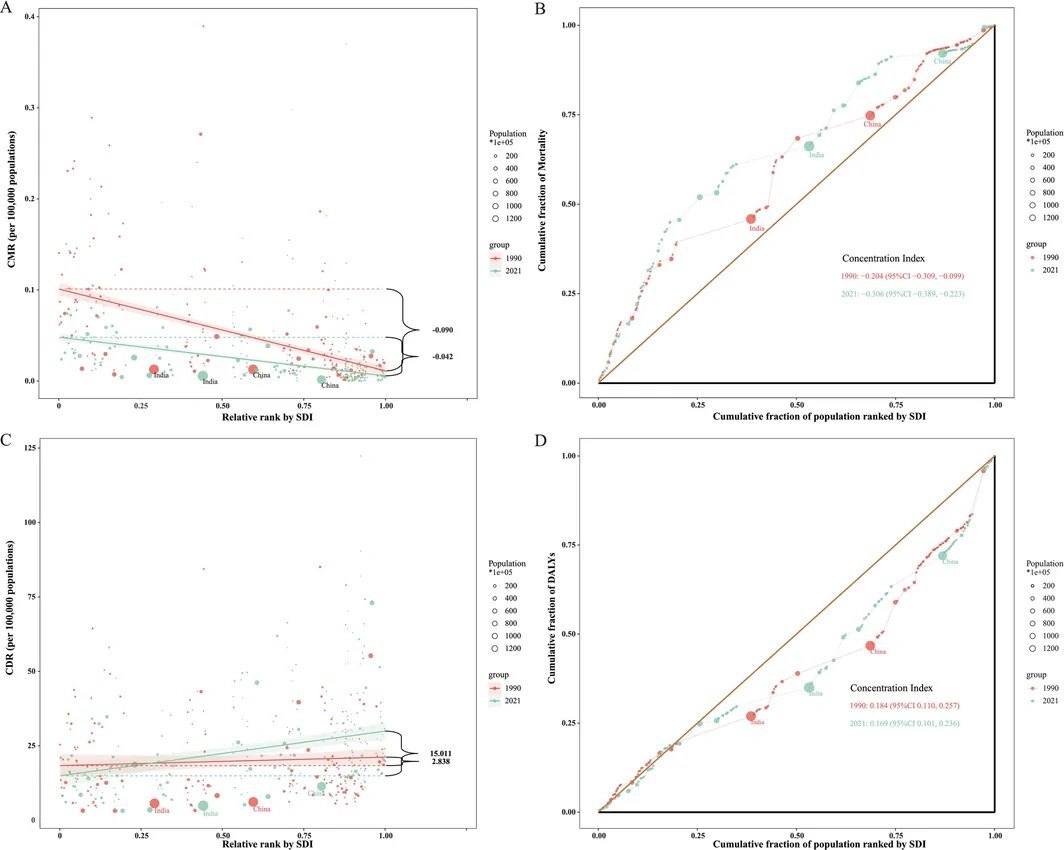
Health inequality regression curves and concentration curves for deaths and DALYs due to asthma attributable to high BMI in children in 1990 and 2021. (A) Regression curve (deaths). (B) Concentration curve (deaths). (C) Regression curve (DALYs). (D) Concentration curve (DALYs). (A and C) Displays the inequality slope index, where each point represents countries and regions stratified by population size. (B and D) Present the concentration index, which quantifies relative inequality by calculating the area under the Lorenz curve. Red indicates data from 1990, while green represents 2021 data. CDR, crude DALYs rate; CMR, crude mortality rate; DALYs, disability‐adjusted life years; SDI, sociodemographic index.

Conversely, CDR burden was predominantly concentrated in high‐SDI countries and regions. The SII rose from 2.838 (95% CI: −2.366 to 8.042) in 1990 to 15.011 (95% CI: 9.296 to 20.727) in 2021, reflecting growing absolute inequality in CDR. Meanwhile, the concentration index declined slightly from 0.184 (95% CI: 0.110–0.257) to 0.169 (95% CI: 0.101–0.236), suggestive of mild attenuation in relative inequality (Figure 5C,D, Table S9).

### 3.6 Frontier Analysis of the Relationship Between Asthma Burden Due to High BMI in Children and SDI

Frontier analyses were performed based on CMR, CDR, and SDI data across the 204 countries and territories from 1990 to 2021. The results showed that several nations, including Somalia, Niger, Bhutan, Cambodia, and Bangladesh, had small efficiency distances in 2021, meaning their CMR values were close to the frontier benchmark. In contrast, Niue, Tokelau, and Kiribati exhibited CMR levels far above the frontier line, indicating substantial room for burden reduction (Figure 6A,B, Table S10). In terms of CDR, low‐SDI countries such as Somalia, Niger, Gambia, Cambodia, and Bangladesh had CDRs relatively near the frontier in 2021. Nevertheless, considerable potential for improvement remained in Puerto Rico, the United States Virgin Islands, Bermuda, and other corresponding territories (Figure 6C,D, Table S10).

**FIGURE 6:**
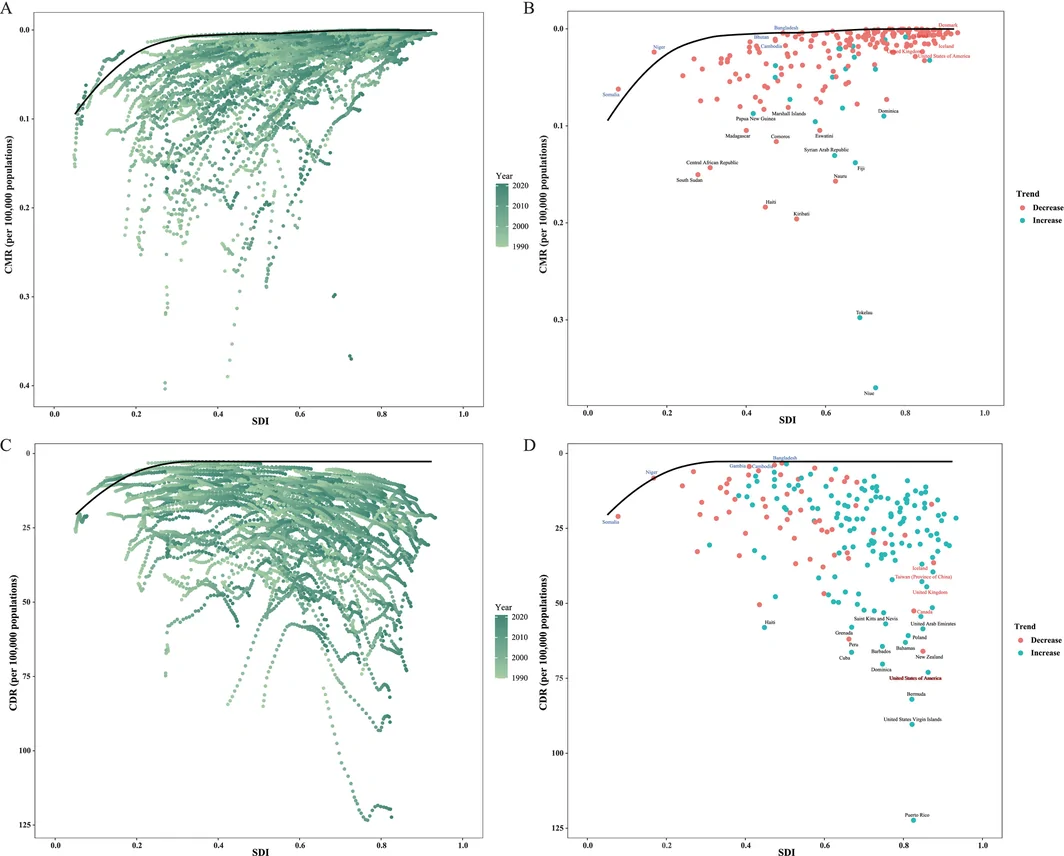
Frontier analysis of the asthma burden attributable to high BMI in children in relation to SDI. (A) Global frontier analysis of CMR versus SDI (1990–2021). (B) Frontier analysis of CMR versus SDI across 204 countries and territories (2021). (C) Global frontier analysis of CDR versus SDI (1990–2021). (D) Frontier analysis of CDR versus SDI across 204 countries and territories (2021). The color gradient represents years from 1990 (light green) to 2021 (dark green). The solid black line demarcates the frontier boundary. Dots represent individual countries and territories. The distance between each dot and the frontier indicates the effective distance. Countries/territories with the largest effective distances (top 15) are labeled in black; Countries/territories with low SDI and minimal effective distances are labeled in blue; Countries/territories with high SDI and relatively large effective distances are labeled in red. CDR, crude DALYs rate; CMR, crude mortality rate; DALYs, disability‐adjusted life years; SDI, sociodemographic index.

### 3.7 Projections Using the BAPC Model for 2022–2035

According to predictions from the BAPC model, the global ASMR of asthma attributable to high BMI among male and female children was projected to keep declining between 2022 and 2035. By 2035, the ASMR was expected to drop to 0.012 per 100,000 (95% CI: 0.003–0.021) for males and 0.015 per 100,000 (95% CI: 0.003–0.026) for females (Figure 7A, Table S11). On the contrary, the ASDRs for both sexes were predicted to maintain an upward trend, reaching 21.659 per 100,000 (95% CI: 8.665–34.653) in males and 18.048 per 100,000 (95% CI: 7.350–28.745) in females by 2035 (Figure 7B, Table S11).

**FIGURE 7:**
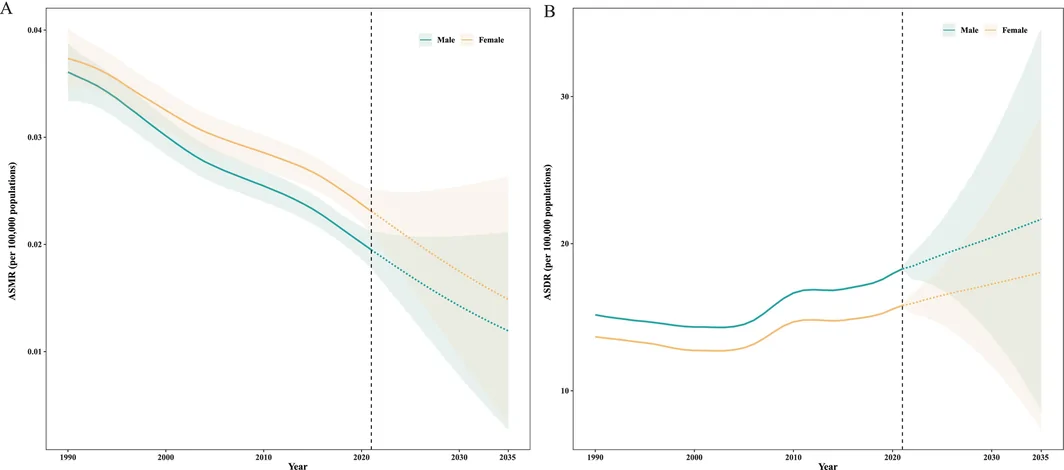
Temporal trends in age‐standardized rates (ASR per 100,000) of deaths and DALYs attributable to high BMI‐related asthma in children globally (2022–2035), stratified by sex. (A) ASMR. (B) ASDR. ASDR, age‐standardized DALYs rate; ASMR, age‐standardized mortality rate; DALYs, disability‐adjusted life years.

## 4 Discussion

Our study demonstrated that from 1990 to 2021, while the global mortality burden of asthma attributable to high BMI in children showed a decreasing trend, the DALYs burden increased. Stratified analyses by sex revealed that females bore a higher mortality burden, while males suffered a greater DALY burden. SDI correlation analysis and cross‐national inequality analysis further revealed that lower‐SDI countries and regions carried higher mortality burdens, while higher‐SDI countries and regions faced greater DALY burdens. Predictive analysis indicates that from 2022 to 2035, asthma mortality burden attributable to high BMI in children will continue to decline, while DALYs burden will maintain an upward trend.

The reduction in mortality burden at the global level may be related to improvements in global healthcare standards, standardized management, and advancements in treatment. Over the past decades, healthcare systems worldwide have progressively enhanced [20]. Moreover, continuously updated global asthma management guidelines, such as the Global Initiative for Asthma (accessed July 8, 2026, https://ginasthma.org/2023‐gina‐main‐report/), combined with the Global Asthma Network [21], have facilitated standardized diagnosis, treatment and patient education for childhood asthma. Therapeutically, beyond the widespread application of inhaled corticosteroids (ICS) and ICS/long‐acting β_2_‐agonists combination therapies (accessed July 8, 2026, https://ginasthma.org/2023‐gina‐main‐report/), the advent of biologics has expanded therapeutic options for children with severe or refractory asthma: omalizumab, mepolizumab, benralizumab, dupilumab, and tezepelumab [22].

However, the DALY burden has become more severe, suggesting that reduced mortality does not equate to diminished disease impact. Adipose accumulation in obese children may restrict thoracic expansion, and such mechanical limitation is linked to constrained ventilation and impaired pulmonary function [23, 24]. Children with higher BMI also tend to consume more high‐fat food, stimulating proinflammatory pathways and increasing bronchial hyperresponsiveness and exacerbating asthma symptoms [25]. In addition, elevated leptin secreted by obesity‐associated adipose tissue can further activate proinflammatory signaling pathways, amplify airway hyperresponsiveness, and induce systemic low‐grade inflammation, thereby driving the development of non‐Th2 or Th2‐low inflammatory phenotypes [24]. Notably, such inflammatory phenotypes exhibit poor responsiveness to ICS therapy [24], which contributes to persistent symptoms and an elevated disease burden.

In terms of stratified analyses by sex, males consistently exhibit higher DALYs burdens than females, while females maintain greater mortality burdens. During childhood, males experience relatively retarded airway development, resulting in relatively narrower airways compared to females [26]. Moreover, males may have a greater susceptibility to asthma allergens. A study found that the prevalence of allergic asthma was higher in male children than females (36.4% vs. 24.0%) [27]. These factors may partly explain the sex disparity in DALY burden. However, obesity may exert a stronger effect on asthma risk in females than in males, as obesity is one factor elevating blood sex hormone levels [28], and increased sex hormones in girls may exacerbate asthma symptoms [29], potentially accounting for the higher mortality burden among females.

Substantial disparities exist in childhood asthma burden across different SDI regions. The increased mortality burden in low‐SDI regions may be related to inadequate healthcare resources and environmental factors. Low‐SDI regions have poorer healthcare accessibility and quality. Although targeted therapies such as biologics have substantially improved clinical outcomes in patients with severe asthma, their widespread use remains limited in many low‐SDI countries and territories due to prohibitive drug costs, insufficient medical insurance coverage in underdeveloped regions, and shortages of specialized medical resources [30], which further widens health inequalities. Furthermore, PM2.5 pollution is more severe in low‐SDI regions, which may also raise the risks of asthma onset and acute exacerbations [31]. In contrast, the DALY burden is more prominent in high‐SDI regions. Epidemiological evidence shows that high‐SDI regions have greater childhood obesity prevalence [32]. The lower mortality rate in such regions allows more children to live with the disease for a long time, thereby increasing the DALY burden.

These findings suggest that differentiated prevention and control strategies should be adopted across regions with varying SDI levels. For lower‐SDI countries, healthcare resources are recommended to be strengthened and investment be increased in primary respiratory care services. Through policy interventions, environmental protection should be enhanced to improve air quality, thereby alleviating childhood asthma burden in low‐SDI regions. High‐SDI countries should prioritize the prevention and standardized management of childhood overweight and obesity, while actively drawing on existing experience. For instance, the soft drinks industry tax implemented in the UK was associated with an approximately 21% reduction in hospital admissions for childhood asthma [33]. Chile's Food Labeling and Advertising Law mandated warning labels on food packaging and restricted the sale of high‐sugar foods and beverages in schools, which led to a roughly 23.7% drop in purchases of sugary drinks [34]. Furthermore, BMI screening and weight management should be incorporated into routine assessments of children with asthma in pediatric clinical practice. Previous research has demonstrated that weight loss improves pulmonary function and asthma control among overweight or obese children [35], indicating that weight management is an essential component of integrated care for pediatric asthma.

This study has several limitations. First, GBD estimates rely on multi‐source data and statistical modeling, and the quality of primary data varies across regions and countries. Especially in areas with limited raw data, model predictions largely depend on covariates and spatiotemporal information, leading to substantial uncertainty in relevant estimates; therefore, these results should be interpreted with caution. Second, the diagnosis of childhood asthma is subject to self‐report bias, limited access to clinical diagnosis, and recall bias. Third, reverse causality may exist between high BMI and asthma: children with severe asthma may reduce physical activity due to exercise‐induced bronchoconstriction or gain weight from long‐term systemic glucocorticoid use. The GBD framework cannot fully eliminate the impact of reverse causality on attributable burden estimates. Fourth, GBD data do not further stratify asthma subtypes, which differ markedly in etiology, pathogenesis, clinical manifestations, treatment responses, and prognoses [36]. Finally, BAPC projections are extrapolated from historical trends and fail to account for potential future effects of obesity prevention strategies and therapeutic advances. For example, semaglutide has demonstrated remarkable efficacy in the treatment of adolescent obesity in recent years [37]. With wider adoption of such interventions, the trajectory of high BMI‐attributable asthma burden among children in the future may deviate from the predictions of this study. However, this example only illustrates that future interventions may cause actual trends to deviate from model predictions, and it does not represent an intervention scenario for model specification or evaluation. Future research based on high‐quality data is warranted to further quantify the effects and temporal trends of high BMI on distinct childhood asthma subtypes and long‐term disease burden.

## 5 Conclusions

From 1990 to 2021, the global mortality burden of childhood asthma attributable to high BMI declined, whereas the DALY burden increased, and this trend was projected to continue from 2022 to 2035. Females bore a higher mortality burden, while males experienced a greater DALY burden. By region, low‐SDI areas carried a heavier mortality burden, and high‐SDI regions showed a more prominent DALY burden. These findings indicate that targeted prevention and control strategies tailored to regional and sex disparities should be implemented to further reduce the burden of asthma attributable to high BMI among children. Low‐SDI regions should strengthen primary‐care capacity for pediatric asthma diagnosis and treatment and improve access to standardized management and essential therapies. By contrast, high‐SDI regions should integrate routine BMI screening and weight management into pediatric asthma follow‐up visits to facilitate early identification and intervention for elevated BMI.

## Conflicts of Interest

The authors declare no conflicts of interest.

## Supporting information


**Table S1:** General cause‐of‐death data‐input star ratings and Percent Well‐Certified‐related indicators for 204 countries and territories, stratified by 2021 sociodemographic index group, 1990–2020.

**Table S2:** Cut‐off values for overweight and obesity in children under 18 years.

**Table S3:** Global burden of asthma attributable to high BMI in children by sex and age group from 1990 to 2021.

**Table S4:** Asthma CMR attributable to high BMI in children and percentage of relative change in 21 regions and 204 countries and territories in 1990 and 2021.

**Table S5:** Asthma CDR attributable to high BMI in children and percentage of relative change in 21 regions and 204 countries and territories in 1990 and 2021.

**Table S6:** EAPC of asthma CMR and CDR attributable to high BMI in children in 21 global regions and 204 countries and territories from 1990 to 2021.

**Table S7:** Trends in asthma CMR and CDR attributable to high BMI in children in relation to SDI globally and in 21 regions from 1990 to 2021.

**Table S8:** Trends in asthma CMR and CDR attributable to high BMI in children in relation to SDI in 204 countries and territories in 2021.

**Table S9:** Health inequality regression curves and concentration curves for asthma deaths and DALYs attributable to high BMI in children in 1990 and 2021.

**Table S10:** Frontier analysis of asthma CMR and CDR attributable to high BMI in children in relation to SDI in 2021.

**Table S11:** Time trends of ASMR and ASDR for asthma attributable to high BMI in children globally among males and females from 2022 to 2035.

## Data Availability

The data that support the findings of this study are available from the corresponding author upon reasonable request.
